# In vitro antioxidant and free radical scavenging activity of different parts of *Tabebuia pallida* growing in Bangladesh

**DOI:** 10.1186/s13104-015-1618-6

**Published:** 2015-10-30

**Authors:** Md. Mahbubur Rahman, Md. Badrul Islam, Mohitosh Biswas, A. H. M. Khurshid Alam

**Affiliations:** Department of Pharmacy, University of Rajshahi, Rajshahi, 6205 Bangladesh; BCSIR, Rajshahi, 6205 Bangladesh

**Keywords:** *Tabebuia pallida*, Free radicals, Polyphenols, Antioxidant

## Abstract

**Background:**

In humans, many diseases are associated with the accumulation of free radicals. Antioxidants can scavenge free radicals and minimize their impact. Therefore, the search for naturally occurring antioxidants of plant origin is imperative. Here, we aimed to investigate the antioxidant and free radical scavenging properties of methanolic extracts from *Tabebuia pallida* (*T. pallida*) stem bark (TPSB), root bark (TPRB), leaves (TPL), and flowers (TPF).

**Methods:**

The antioxidant and free radical scavenging activity were determined by several standard methods using spectrophotomer. Total phenolic and flavonoid contents were estimated using Folin-Ciocalteu reagent and aluminum chloride colorimetric assay methods, respectively.

**Results:**

Among the extracts, TPL showed the highest total antioxidant capacity followed by TPRB, TPF, and TPSB. Based on DPPH and hydroxyl radical scavenging activity, TPL showed strong scavenging activity (91.05 ± 1.10 and 62.00 ± 0.57) with IC_50_ of 9.20 ± 0.28 and 46.00 ± 2.84 μg/mL, respectively when compared with standard BHT (IC_50_ of 7.00 ± 0.25 μg/mL) and CA (75.00 ± 0.14 μg/mL). These results suggest that TPL had the highest radical scavenging activity among the extractives that closely resembled the standard’s. In lipid peroxidation inhibition assay, TPL exhibited the most potent inhibitory activity (83.18 ± 2.12 %) with IC_50_ of 12.00 ± 2.12 μg/mL, which closely resembled standard CA (IC_50_ of 10.50 ± 0.28 μg/mL). Also, the reducing capacity on ferrous ion was in the following order: TPL > TPRB > TF > TPSB. The phenolic and flavonoid contents of TPL were higher than other extractives. A positive correlation (*pvalue* <*0*.001) was observed between phenolic content and free radical (DPPH^·^ and ^·^OH) scavenging efficiencies and lipid peroxidation inhibition activity.

**Conclusion:**

Methanolic extract of *T. pallida* leaf is a potential source of natural antioxidants and serves as an effective free radical scavenger and/or inhibitor. Hence, *T. pallida* might be a good plant-based pharmaceutical product for several diseases caused by free radicals.

**Electronic supplementary material:**

The online version of this article (doi:10.1186/s13104-015-1618-6) contains supplementary material, which is available to authorized users.

## Background

There is strong evidence that many dangerous pathophysiological processes, such as cancer, diabetes and cardiovascular and neurodegenerative diseases, are associated with the accumulation of free radicals [1–3]. A free radical is an atom or molecule that has an unpaired electron and is therefore unstable. This unstable radical has the tendency to become stable through electron pairing with biological macromolecules such as proteins, lipids, and DNA in healthy human cells, thus causing protein and DNA damage [1]. Such radical-caused cell damage can become more widespread due to weakened cellular antioxidant defense systems. All biological systems have innate antioxidant defense mechanisms that remove damaged molecules, but these mechanisms can be inefficient. Therefore dietary intake of antioxidants is imperative to protect cells from damage caused by free radicals.

Antioxidants are substances that prevent and stabilize the damage caused by free radicals by supplying electrons from antioxidants to these damage cells. Antioxidants also turn free radicals into waste by-products, which are eliminated from the body. Consumption of antioxidant-enriched fruits and vegetables is known to lower the risk of several diseases caused by free radicals [4]. Such health benefits are mainly due to the presence of phytochemicals such as polyphenols, carotenoids, and vitamin E and C [5]. Although phenolic compounds are commonly found in both edible and in non edible herbs, cereals, fruits, vegetables, oils, spices, and other plant materials [6, 7], scientific information on the antioxidant properties of endemic plants is scare because the availability of endemic plants is limited to certain regions and only known by local populations. Therefore, the assessment of such properties remains an interesting and useful task, particularly to find promising sources of natural antioxidants for functional foods and/or nutraceuticals [7, 8].

*Tabebuia palllida* (commonly known as White trumpet tree), is one of the largest genera of the Bignoniaceae family distributed in central America, West India, and South America [9, 10]. To the best of our knowledge, there is no work investigating the phytochemical composition and biological activities of *T. palllida*. Therefore, we performed a detailed literature review of the *Tabebuia* genus. We found that *Tabebuia* has a variety of biological activities [9] and is recognized as a therapeutic alternative by rural or remote populations for the treatment of different diseases. *Tabebuia rosea* is used as an antipyretic, anti-inflammatory, antibacterial, antifungal, anti-cancer, and anti-diabetic agent [10–15]. Stem bark of *T. avellanedae* is used in the treatment of snake bites [10, 16]. Also, *T. heterophylla*, *T. aurea*, *T. argentea* and *T. caraiba* are used as anticancer, anti-inflammatory, and antimicrobial agents [9, 17].

The results of ethnobotanical and ethnopharmacological studies of different species of *T. pallida* indicate the potential use of these plants for the treatment of a large variety of diseases. Due to the increasing interest in the relationship between antioxidants and diseases, it is important to measure the overall antioxidant activity of *T. pallida*. Therefore, the objective of this study was to evaluate the antioxidant and free radical scavenging activity as well as polyphenol contents of methanolic extractives from different parts of *T. pallida*.

## Methods

### Plant collection

Leaves, flowers, stem and root barks of *T. pallida* were collected from Rajshahi University Campus, Rajshahi, Bangladesh on 11th May, 2013 and were identified by an expert taxonomist at the Department of Botany, University of Rajshahi, where a voucher specimen (Voucher No. MN-03) was deposited. Plant materials were then washed separately with fresh water to remove dirt and other contaminants, and were shade-dried for several days with occasional sun drying. The dried materials were ground into coarse powder by a grinding machine and the materials were stored at room temperature (RT) for future use.

### Preparation of the extract

The extraction was performed according to Alam et al. [18]. About 500 g of each powdered plant materials was taken in four amber colored extraction bottles and soaked with 1.5 L of methanol. The sealed bottles were kept for 15 days with occasional shaking and stirring. The extracts were filtered separately through a fresh cotton plug and finally with Whatman No.1 filter papers. The filtrates were concentrated with a rotary evaporator (Bibby Sterlin Ltd, UK) under reduced pressure at 50 °C to afford 30, 35, 45 and 40 g extract of leaves, flowers, stem and root barks extract, respectively.

### Chemicals

1,1-diphenyl-2-picrylhydrazyl (DPPH), potassium ferricyanide, catechin (CA), ferrous ammonium sulphate, butylated hydroxytoluene (BHT), gallic acid (GA), ascorbic acid (AA), AlCl_3_, trichloro acetic acid (TCA), sodium phosphate, sodium nitrate, ammonium molybdate, sodium hydroxide, EDTA and FeCl_3_ were purchased from Sigma Chemical Co. (St. Louis, MO, USA); potassium chloride, potassium acetate, phosphate buffer, 2-deoxy-d-ribose, thiobarbituric acid (TBA), HCl, H_2_SO_4_, H_2_O_2_ were purchased from Sigma-Aldrich, folin-ciocalteus’s phenol reagent and sodium carbonate were obtained from Merck (Dam-stadt, Germany).

### Determination of total phenolics

Total phenolic contents in the extracts were determined by the modified Folin-Ciocalteu method described by Wolfe et al. [19]. An aliquot of the extract was mixed with 2 mL Folin-Ciocalteu reagent (previously diluted with water 1:10 v/v) and 2 mL (75 g/L) of sodium carbonate. The tubes were vortexed for 15 s and allowed to stand for 20 min at 25 °C for color development. Absorbance was then measured at 760 nm UV-spectrophotometer (Shimadzu, USA). Samples of extract were evaluated at a final concentration of 0.1 and 0.15 mg/mL. Total phenolic contents were expressed in terms of gallic acid equivalent, GAE (standard curve equation: y = 0.091× + 0.167, R^2^ = 0.994), mg of GA/g of dry extract. The experiment was repeated three times at each concentration.

### Determination of total flavonoids

Total flavonoids were estimated using aluminum chloride colorimetric assay described by Zhisen et al. [20]. To 0.5 mL of samples/standard, 150 µL of 5 % sodium nitrate and 2.5 mL of distilled water were added. After 5 min, 0.3 mL of 10 % AlCl_3_ was added. At 6 min, 1 mL of 0.001 M NaOH and 0.55 mL distilled water was added to the mixture and left at RT for 15 min. Absorbance of the mixtures was measured at 510 nm. Samples of extract were evaluated at a final concentration of 0.1 and 0.15 mg/mL. Total flavonoid contents were expressed in terms of catechin equivalent, CAE (standard curve equation: y = 0.000× + 0.001, R^2^ = 0.998), mg of CA/g of dry extract. The experiment was repeated three times at each concentration.

### Determination of total antioxidant capacity

Total antioxidant capacity (TAC) of samples was determined by the method reported by Prieto et al. [21]. The assay is based on the reduction of Mo(VI) to Mo(V) by samples and formation of green colored phosphate/Mo(V) complex at acidic pH. 0.5 mL of samples/standard at different concentrations (12.5–150 μg/mL) was mixed with 3 mL of reaction mixture containing 0.6 M sulphuric acid, 28 mM sodium phosphate and 1 % ammonium molybdate into the test tubes. The test tubes were incubated at 95 °C for 10 min to complete the reaction. After cooling at RT, sample absorbance was measured at 695 nm using a spectrophotometer against a blank solution. CA was used as standard. A typical blank solution contained 3 mL of reaction mixture and the appropriate volume of the same solvent used for the samples/standard. The blank was incubated at 95 °C for 10 min and the absorbance was measured at 695 nm. Increased absorbance of the reaction mixture indicates increased total antioxidant capacity. We used standard/samples at five different concentrations ranges from 12.5 to 150 μg/mL for each antioxidant assay. Concentrations were selected on the basis of trial and error to fit the range of concentration that can fully represent the rational change of antioxidant activity with the increasing concentration of the samples. Also, we assumed that such range of concentrations is useful for smooth calculation of IC_50_. The experiment was repeated three times at each concentration.

### Ferrous reducing antioxidant capacity assay

The ferrous reducing antioxidant capacity (FRAC) of samples was evaluated by the method of Oyaizu [22]. The Fe^2+^ can be monitored by measuring the formation of Perl’s Prussian blue at 700 nm. 0.25 mL samples/standard solution at different concentration (12.5–150 μg/mL), 0.625 mL of potassium buffer (0.2 M) and 0.625 mL of 1 % potassium ferricyanide, [K_3_Fe (CN)_6_] solution were added into the test tubes. The reaction mixtures were incubated for 20 min at 50 °C to complete the reaction. Then 0.625 mL of 10 % trichloro acetic acid (TCA) solution was added into the test tubes. The total mixture was centrifuged at 3000 rpm for 10 min, after which 1.8 mL supernatant was withdrawn from the test tubes and mixed with 1.8 mL of distilled water and 0.36 mL of 0.1 % ferric chloride (FeCl_3_) solution. The absorbance of the solution was measured at 700 nm using a spectrophotometer against blank. A typical blank solution contained the same solution mixture without plant extracts/standard and was incubated under the identical conditions. The absorbance of the blank solution was measured at 700 nm. Increased absorbance of the reaction mixture indicates increased reducing capacity. The experiment was repeated three times at each concentration.

### DPPH radical scavenging assay

Free radical scavenging ability of the extracts was tested by DPPH radical scavenging assay as described by Blois [23] and Desmarchelier et al. [24]. The hydrogen atom donating ability of the plant extractives was determined by the decolorization of methanol solution of 2,2-diphenyl-1-picrylhydrazyl (DPPH). DPPH produces violet/purple color in methanol solution and fades to shades of yellow color in the presence of antioxidants. A solution of 0.1 mM DPPH in methanol was prepared, and 2.4 mL of this solution was mixed with 1.6 mL of extract in methanol at different concentrations (12.5–150 μg/mL). The reaction mixture was vortexed thoroughly and left in the dark at RT for 30 min. The absorbance of the mixture was measured spectrophotometrically at 517 nm. BHT was used as reference. Percentage DPPH radical scavenging activity was calculated by the following equation:

$$\% {\text{ DPPH radical scavenging activity}} = \left\{ {\left( {{\text{A}}_{0} {-}{\text{ A}}_{ 1} } \right)/{\text{A}}_{0} } \right\} \times 100$$

where A_0_ is the absorbance of the control, and A_1_ is the absorbance of the extractives/standard. Then  % of inhibition was plotted against concentration, and from the graph IC_50_ was calculated. The experiment was repeated three times at each concentration.

### Hydroxyl radical scavenging activity

Hydroxyl radical scavenging activity of the extractives was determined by the method of Halliwell et al. [25]. Hydroxyl radical was generated by the Fe^3+^-ascorbate-EDTA-H_2_O_2_ system (Fenton reaction). The assay is based on the quantification of the 2-deoxy-d-ribose degradation product, which forms a pink chromogen upon heating with TBA at low pH. The reaction mixture contained 0.8 mL of phosphate buffer solution (50 mmol L^−1^, pH 7.4), 0.2 mL of extractives/standard at different concentration (12.5–150 μg/mL), 0.2 mL of EDTA (1.04 mmol L^−1^), 0.2 mL of FeCl_3_ (1 mmol L^−1^) and 0.2 mL of 2-deoxy-d-ribose (28 mmol L^−1^). The mixtures were kept in a water bath at 37 °C and the reaction was started by adding 0.2 mL of ascorbic acid, AA (2 mmol L^−1^) and 0.2 mL of H_2_O_2_ (10 mmol L^−1^). After incubation at 37 °C for 1 h, 1.5 mL of cold thiobarbituric acid, TBA (10 g L^−1^) was added to the reaction mixture followed by 1.5 mL of HCl (25 %). The mixture was heated at 100 °C for 15 min and then cooled down with water. The absorbance of solution was measured at 532 nm with a spectrophotometer. The hydroxyl radical scavenging capacity was evaluated with the inhibition of percentage of 2-deoxy-d-ribose oxidation on hydroxyl radicals. The percentage of hydroxyl radical scavenging activity was calculated according to the following formula:

$$\% {\text{ hydroxyl radical scavenging activity}} = [{\text{A}}_{0} {-} \, \left( {{\text{A}}_{ 1} {-}{\text{A}}_{ 2} } \right] \times 100/{\text{A}}_{0}$$

where A_0_ is the absorbance of the control without a sample. A_1_ is the absorbance after adding the sample and 2-deoxy-D-ribose. A_2_ is the absorbance of the sample without 2-deoxy-d-ribose. Then  % of inhibition was plotted against concentration, and from the graph IC_50_ was calculated. The experiment was repeated three times at each concentration.

### Lipid peroxidation inhibition assay

The lipid peroxidation inhibition assay was determined according to the method described by Haenen and Bast [26]. Protocol used in this study for the use of rat as animal model for lipid peroxidation inhibition assay was approved by the Institutional Animal, Medical Ethics, Biosafety and Biosecurity Committee (IAMEBBC) for Experimentations on Animal, Human, Microbes and Living Natural Sources (225/320-IAMEBBC/IBSc), Institute of Biological Sciences, University of Rajshahi, Bangladesh. Excised rat liver was homogenized in buffer and then centrifuged to obtain liposome. To make 10 % liver homogenate, excised Wister rat liver (weight of ~150 g) was minced using glass Teflon homogenizer in ice cold phosphate buffered saline (50 mm, pH 7.4). The homogenate was centrifuged at 12,000 rpm for 15 min at 4 °C. The supernatant was used as liposome for in vitro lipid peroxidation assay. The process of homogenization and filtration was carried out in ice cold condition. Firstly, 0.5 mL of supernatant, 1 mL of 0.15 M KCl, and 0.3 mL of extractives or standard at different concentrations (12.5–150 μg/mL) were mixed. Peroxidation was initiated by the addition of 300 μL of 0.5 mM FeCl_3_. The mixture was incubated at 37 °C for 30 min and the reaction was stopped by adding 2 mL of ice-cold TBA-TCA-HCl-BHT solution. The TBA-TCA-HCl solution was prepared by dissolving 1.68 mg TCA and 41.60 mg TBA in 10 ml of 0.125 M HCl. 1 mL BHT solution (1.5 mg/mL ethanol) was added to 10 mL TBA-TCA HCl solution. The reaction mixture was heated for 60 min at 90 °C and then cooled on ice and centrifuged at 3000 rpm for 5 min. The supernatants were removed and the intensity of the pink colored complex was measured using a spectrophotometer at 532 nm. The degree of lipid peroxidation was assayed by estimating the TBARS (TBA-reactive substances) content. A control experiment was performed in the presence of distilled water without the extract. The percentage of lipid peroxidation inhibition in the samples was calculated using the following formula:

$$\% {\text{ lipid peroxidation inhibition}} = \left[ {\left( {{\text{A}}_{0} {-}{\text{A}}_{ 1} } \right)/{\text{A}}_{0} } \right] \times 100$$

where A_0_ is the absorbance of the control (300 μl of distilled water), and A_1_ is the absorbance of extractives/standard. Then  % of inhibition was plotted against concentration, and from the graph IC_50_ was calculated. The experiment was performed in triplicate and repeated three times at each concentration.

### Statistical analysis

All tests were carried out in triplicates. Data were presented as mean ± SD. To evaluate significant relationships between experimental parameters by correlation and regression analysis, the F- and t-tests (p-value <0.001) were used. Free R-software version 2.15.1 (http://www.r-project.org/) and Microsoft Excel 2007 (Roselle, IL, USA) were used for the statistical and graphical evaluations.

## Results

### Determination of TAC and FRAC

The TAC and FRAC of methanolic extracts of different parts of *T.Pallida* are shown in Table 1. Methanolic extracts of different parts of *T.Pallida* showed considerable antioxidant activity when compared with standard CA. At the concentration of 100 μg/mL, the absorbance of methanolic extracts of TPL, TPRB, TPSB, TPF and CA was in the range of 0.525 ± 0.032–1.78 ± 0.083; while at 150 μg/mL, the absorbance of methanolic extracts of TPL, TPRB, TPSB, TPF and CA was in the range of 0.648 ± 0.030-2.267 ± 0.041. Increasing the extractives’ concentration increased the total antioxidant activity.

**Table 1:** Absorbance of TAC and FRAC of different parts (TPL, TPRB, TPSB and TPF) of *T. pallida* at two different concentrations (n = 3, X ± SEM)

Extractives	TAC		FRAC	
	At 100 µg/mL	At 150 µg/mL	At 100 µg/mL	At 150 µg/mL
TPL	1.256 ± 0.026^a^	1.625 ± 0.010	2.199 ± 0.135	2.914 ± 0.100
TPRB	0.912 ± 0.097	1.150 ± 0.073	0.503 ± 0.002	0.741 ± 0.030
TPSB	0.685 ± 0.099	0.836 ± 0.063	0.289 ± 0.057	0.484 ± 0.028
TPF	0.525 ± 0.032	0.648 ± 0.030	0.454 ± 0.029	0.669 ± 0.094
CA	1.78 ± 0.083	2.267 ± 0.041	–	–
AA	–	–	3.108 ± 0.069	3.223 ± 0.087

TPL, TPRB, TPSB and TPF are representing as *T. pallida* leaf, root bark, stem bark and flower, respectively. AA and CA are representing as ascorbic acid and catechin, respectively

^**a**^Each value is the average of three analyses ± standard deviation

The methanolic extracts of *T. pallida* showed moderate to high FRAC with increased concentration of the extracts. At 100 µg/mL, the absorbance of methanolic extracts of TPL, TPRB, TPSB, TPF and AA was in the range of 0.289 ± 0.057–3.108 ± 0.069; whereas at 150 µg/mL, the absorbance of methanolic extracts of TPL, TPRB, TPSB, TPF and AA was in the range of 0.484 ± 0.028–3.223 ± 0.087. A higher absorbance indicates a higher reducing power. These results demonstrated that the methanolic extract of TPL possessed the highest TAC and FRAC among all other extractives, closely resembling that of standards CA and AA (Table 1).

### DPPH radical scavenging activity

**Fig. 1:**
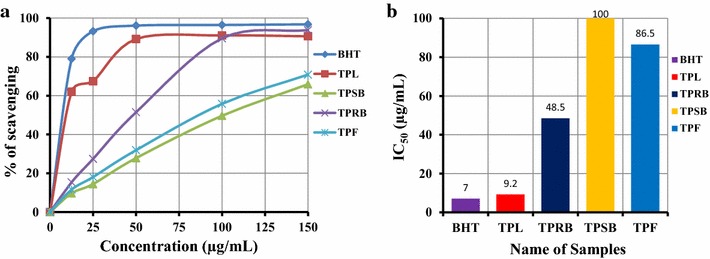
Determination of **a** DPPH radical scavenging activity and **b** IC_50_ of methanolic extractives (TPL, TPRB, TPSB and TPF) of *T. pallida*. All experiments were performed in triplicate. Data are expressed as mean ± SD (n = 3, p < 0.05) for all tested dosages

Figure 1a shows the free radical scavenging activity of the methanolic extracts of TPL, TPRB, TPSB, and TPF of *T. pallida* and standard BHT. Among the extractives, TPL possessed the highest activity. At a concentration of 100 μg/mL, the scavenging activity of TPL, TPRB, TPSB, and TPF was 91.05 ± 1.10, 89.55 ± 0.70, 49.65 ± 1.95 and 55.85 ± 0.30 %, respectively, whereas at the same concentration, the standard BHT was 96.45 ± 0.41 % (Fig. 1a). The IC_50_ of methanolic extracts of TPL, TPRB, TPSB, and TPF was 9.2 ± 0.28, 48.5 ± 1.70, 100 ± 4.66 and 86.5 ± 1.04 μg/mL, respectively. The IC_50_ of BHT (standard) was 7.00 ± 0.25 μg/mL (Fig. 1b). The free radical scavenging activity of different extracts and BHT was in the following order: BHT > TPL > TPRB > TPF > TPSB.

### Hydroxyl radical scavenging activity

**Fig. 2:**
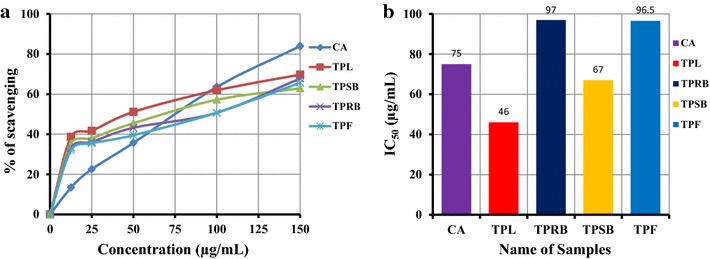
Determination of **a** Hydroxyl radical scavenging activity and **b** IC_50_ of methanolic extractives (TPL, TPRB, TPSB and TPF) of *T. pallida* plant. All experiments were performed in triplicate. Data are expressed as mean ± SD (n = 3, p < 0.05) for all tested dosages

The hydroxyl radical scavenging activity of the methanolic extracts of TPL, TPRB, TPSB, and TPF was dose dependent. Among the extracts, TPL had higher activity than that of the other extracts. At a concentration of 100 µg/mL, the scavenging activity of TPL, TPRB, TPSB and TPF was 62.00 ± 0.57, 50.64 ± 0.08, 57.21 ± 0.16 and 50.70 ± 0.33 %, respectively, whereas at the same concentration, CA’s was 63.59 ± 0.23 % (Fig. 2a). The hydroxyl radical scavenging activity of TPL was approximately equal to that of CA (standard). The IC_50_ of TPL, TPRB, TPSB, TPF and CA was 46.00 ± 2.84, 97.00 ± 0.86, 67.5 ± 1.25, 96.5 ± 1.28 and 75.00 ± 0.14 µg/mL, respectively, demonstrating that the inhibitory activity of TPL was higher than standard CA (Fig. 2b).

### Lipid peroxidation inhibition assay

**Fig. 3:**
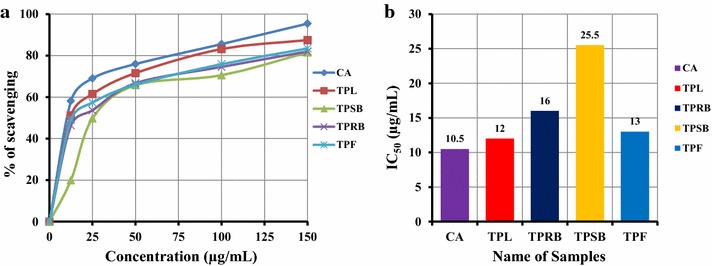
Determination of **a** Lipid peroxidation inhibition activity and **b** IC_50_ of methanolic extractives (TPL, TPRB, TPSB and TPF) of *T. pallida*. All experiments were performed in triplicate. Data are expressed as mean ± SD (n = 3, p < 0.05) for all tested dosages

The lipid peroxidation inhibition activity of TPL, TPRB, TPSB and TPF was compared with CA. At a concentration of 100 µg/mL, the inhibitory activity of TPL, TPRB, TPSB and TPF was 83.18 ± 0.33, 74.56 ± 0.45, 70.53 ± 0.55 and 75.88 ± 0.21 %, respectively; whereas that of the CA was 85.53 ± 0.49 % (Fig. 3a). The IC_50_ of TPL, TPRB, TPSB, TPF and CA were 12.00 ± 0.14, 16.00 ± 0.57, 25.50 ± 0.50, 13 ± 1.08 and 10.50 ± 0.28 μg/mL, respectively (Fig. 3b). TPL had higher inhibitory activity than other extracts and almost equal inhibitory activity to that of CA. A positive correlation (*p*-*value* <*0.001*) of lipid peroxidation inhibition with free radical (DPPH^·^ and ^·^OH) scavenging activities was observed.

### Total phenolic and flavonoid contents

Table 2 shows the total polyphenols contents in the methanolic extracts of TPL, TPRB, TPSB, and TPF expressed as GAE and CAE. A standard curve for total phenolic and flavonoid contents is shown in Additional file 1: Figure S1 (A, B). A positive correlation (*p*-*value* <*0.001*) of total phenolic content of the extractives with free radical (DPPH^·^ and ^·^OH) scavenging efficiencies and  % of lipid peroxidation inhibition was observed (Additional file 2: Table S1).

**Table 2:** Polyphenol contents of the methanolic extracts of TPL, TPRB, TPSB, and TPF (n = 3, X ± SEM)

Polyphenols	TPL	TPRB	TPF	TSB
Phenolics^*a*^	56.19 ± 1.11^1^	12.81 ± 0.544	15.93 ± 0.974	12.71 ± 0.890
Flavonoids^*b*^	0.55 ± 0.02	0.16 ± 0.016	0.24 ± 0.025	0.23 ± 0.030

*a* and *b* expressed in terms of GAE and CAE, respectively (mg of GA and CA/g of dry extract, respectively)

^1^Each value is the average of three analyses ± standard deviation

## Discussion

For assessment of antioxidant potential of endogenous compounds, a single assay method is not sufficient. Furthermore, different antioxidant assays vary in terms of assay principle and experimental conditions. For instant, some methods use organic radical producers e.g. DPPH and some use metal ions for oxidation e.g. FRAC assay technique. The time factor associated with their chemical reactions to produce free radicals by oxidation reaction also different from each other. Since the procedure and experimental conditions are different for different techniques, the various antioxidants are considered as a control for different assay techniques according to their rate and time of scavenging. In addition, antioxidants could be polar e.g. phenolics, flavanoids etc. or non-polar e.g. vitamin E in nature and they can act as radical scavenger by electron donating mechanism or by hydrogen donating mechanism. Therefore, different control antioxidants (e.g. BHT, AA, CA) were used for different antioxidant assays.

### Total antioxidant capacity and ferrous reducing antioxidant activity

The antioxidant potential of the different parts of methanolic extracts of TPL, TPRB, TPSB, and TPF of *T. pallida* was estimated from their ability to reduce the reduction of Mo (VI) to Mo (V) by the antioxidant-enriched fractions and subsequent formation of a green phosphate/Mo (V) complex at acidic pH. Antioxidant activity depends on the presence of its bio-active compounds mainly polyphenols, carotenoids, and vitamin E and C (27). This suggests that the concentration of the bioactive compounds present in the extract is important to showing antioxidant activity. Thus, higher concentration of extracts shows higher antioxidant activity. In this study, the reducing ability of the extractives was in the range of 0.648 ± 0.030–1.625 ± 0.010 μm green phosphate/Mo (V). All the extracts showed a good total antioxidant activity that increased with increasing concentration (Table 1). Our results comply with the data published elsewhere [27] and suggest that the antioxidant capacity can be attributed to the extractive’s chemical composition and polyphenol contents.

Reducing power is also widely used in evaluating antioxidant activity of plant polyphenols. The reducing power is generally associated with the presence of reductants, which exert antioxidant action by breaking the free radical chains by donating a hydrogen atom. In this assay, the presence of reductants in the antioxidant sample reduces Fe^3+^/ferricyanide complex to the Fe^2+^/ferrous form. Thus, the reducing power of the sample can be monitored by measuring the formation of Perl’s Prussian blue at 700 nm [27]. In this study, the iron reducing capacity of the methanolic extracts of TPL, TPRB, TPSB, and TPF was estimated from their ability to reduce the Fe^3+^-ferricyanide complex to the ferrous form by donating an electron. The reducing ability of the extracts was in the range of 0.484 ± 0.028–2.914 ± 0.100 μm Fe(II)/g (Table 1). All the extracts showed a good reducing power capacity, which was concentration-dependent (Table 1). Our results are consistent with the data published previously [28]. Here, we assume that the antioxidant activity and reducing power capacity of the extracts was likely due to the presence of polyphenols, which can act as free radicals scavenger by donating an electron or hydrogen.

### DPPH radical scavenging activity

The effect of antioxidants on DPPH is thought to be due to their hydrogen donating ability [29]. Radical scavenging activities are very important to prevent the deleterious role of free radicals in different diseases, including cancer. DPPH free radical scavenging is an accepted mechanism for screening the antioxidant activity of plant extracts. In the DPPH assay, violet color DPPH solution is reduced to yellow colored product, diphenylpicryl hydrazine, by the addition of the extract in a concentration dependent manner. This method has been used extensively to predict antioxidant activities because of the relatively short time required for analysis. Our results revealed that the methanolic extract of TPL had a similar free radical scavenging activity when compared with standard BHT (Fig. 1). Polyphenol contents and tocopherols scavenge the DPPH radicals by their hydrogen donating ability [28, 29]. The results obtained in this study suggest that all the extracts from *T. pallida* showed radical scavenging activity by their electron transfer or hydrogen donating ability. Total polyphenols content and radical scavenging antioxidant activity are highly correlated [28].

### Hydroxyl radical scavenging activity

The mutagenic capacity of free radicals is due to the direct interactions of hydroxyl radicals with DNA, resulting in DNA breakdown and therefore playing an important role in cancer formation [30]. Hydroxyl radicals are formed by incubating Fe^+3^- EDTA premixture with ascorbic acid and H_2_O_2_ at pH 7.4, causing 2-deoxy-d-ribose degradation and generating a malondialdehyde (MDA)-like product. Addition of the methanolic extractives of TPL, TPRB, TPSB, and TPF to the reaction mixture removes hydroxyl radicals and prevents further damage. The extractives showed appreciable hydroxyl radical scavenging activity when compared with standard antioxidant, CA (Fig. 2), and could be served as anticancer agents by inhibiting the interaction of hydroxyl radicals with DNA. The ability of the extractives to quench hydroxyl radicals might directly relate to the prevention of lipid peroxidation.

### Lipid peroxidation inhibition assay

Reactive oxygen species induce membrane damage by peroxiding lipid moieties, particularly the polyunsaturated fatty acids in a chain reaction known as lipid peroxidation [31]. The initial reaction generates a second radical, which can further react with a second macromolecule, generating chain reaction and causing cellular abnormalities. In vitro lipid peroxidation was induced in rat liver by ferric ion plus potassium chloride through OH radical generation by Fenton’s reaction. The inhibition of lipid peroxidation is considered the most important index of antioxidant activity. Here, lipid peroxidation inhibition activity of TPL was higher than other extractives (Fig. 3). These results indicated that *T. pallida* can prevent cellular abnormalities caused by free radicals by breaking down the chain reactions responsible for lipid peroxidation. Thus, *T. pallida* is a good source of natural antioxidants and may be used to treat several diseases caused by free radicals.

### Total phenolic and flavonoid contents

Total phenolic content of the extractives showed significant and strong positive correlation (*p*-*value* <*0*.001) with free radical (DPPH^·^ and ^·^OH) scavenging efficiencies and  % of lipid peroxidation inhibition. The R^2^ values (correlation coefficients) between phenolic contents and free radical scavenging efficiencies and lipid peroxidation inhibition were shown in Additional file 2: Table S1. Our results are consistent with the data published previously [3, 32, 33]. Thus, the polyphenolic constituents of the extracts may be the major contributors to the antioxidant activity in free radical neutralization and lipid peroxidation inhibition.

## Conclusion

Biologically active pure compound is better than crude extract. However, to get the overall view regarding the phytochemical composition and biological activities of any plant, it is important to pick the most potential part by investigating several parts (leaf, stem bark, root bark, fruits, flowers etc.) of that plant. Here, we examined different parts (root bark, stem bark, leaf and flower) of *T. pallida* and found that methanolic extract of *T. pallida* leaf, which contains large amounts of phenolic and flavonoid compounds, exhibited the highest antioxidant and free radical scavenging and also inhibited lipid peroxidation. A positive correlation (*p*-*value* <*0*.001) was observed between phenolic content and free radical (DPPH^·^ and ^·^OH) scavenging efficiencies and lipid peroxidation inhibition activity. These in vitro assays indicate that *T. pallida* leaves are a significant source of natural antioxidants, which could help to prevent the progression of various diseases caused by free radicals, such as certain cancers. However, the components responsible for the antioxidative activity are currently unclear. Therefore, further investigation is needed to isolate and identify the antioxidant compounds present in the plant extract.

## Additional files

10.1186/s13104-015-1618-6 Standard curves for (A) phenolic and (B) flavonoid contents.
10.1186/s13104-015-1618-6 The R^2^ values (correlation coefficients) between phenolic contents and free radical scavenging efficiencies and lipid peroxidation inhibition.

## Abbreviations

AA: ascorbic acid
CA: catechin
CAE: catechin equivalent
DPPH: 1,1-diphenyl-2-picrylhydrazyl
EDTA: ethylenediamine tetraacetic acid
BHT: butylated hydroxytoluene
FRAC: ferrous reducing antioxidant capacity
GA: gallic acid
GAE: gallic acid equivalent
TAC: total antioxidant capacity
TCA: trichloro acetic acid
TBA: thiobarbituric acid
TPL: *Tabebuia pallida* leaves
TPF: *Tabebuia pallida* flower
TPRB: *Tabebuia pallida* root bark
TSB: *Tabebuia pallida* stem bark
