# Supplementary figures and images for: In vitro antioxidant and free radical scavenging activity of different parts of Tabebuia pallida growing in Bangladesh
Source: BMC Res Notes. 2015 Oct 30;8:621. doi: 10.1186/s13104-015-1618-6 (PMC4627625; doi:10.1186/s13104-015-1618-6)

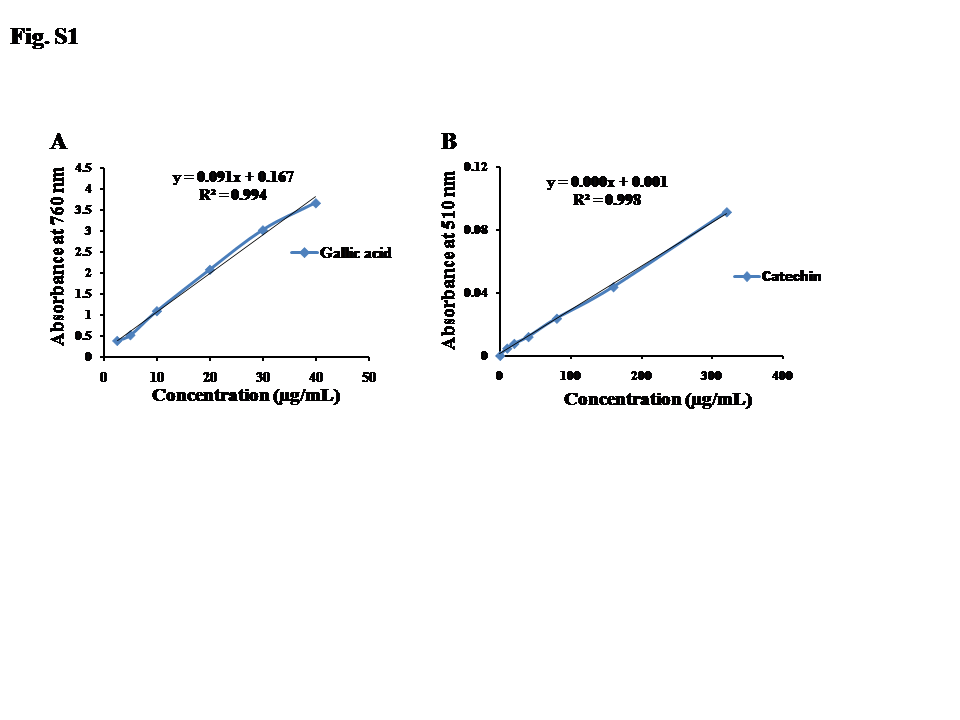

Supplement: Supplementary file 1 — 10.1186/s13104-015-1618-6 Standard curves for (A) phenolic and (B) flavonoid contents. [file 13104_2015_1618_MOESM1_ESM.tiff]
