# Supplementary material for: In vitro antioxidant and free radical scavenging activity of different parts of Tabebuia pallida growing in Bangladesh
Source: BMC Res Notes. 2015 Oct 30;8:621. doi: 10.1186/s13104-015-1618-6 (PMC4627625; doi:10.1186/s13104-015-1618-6)
Supplement: Supplementary file 2 — 10.1186/s13104-015-1618-6 The R2 values (correlation coefficients) between phenolic contents and free radical scavenging efficiencies and lipid peroxidation inhibition. [file 13104_2015_1618_MOESM2_ESM.doc]

**Additional file 2: Table S1:** The R^2^ values (correlation coefficients) between phenolic contents and free radical scavenging efficiencies and lipid peroxidation inhibition.

|  | DPPH | Hydroxyl radical | Lipid peroxidation |
| --- | --- | --- | --- |
| Total phenolic | R^2^ =0.96±0.020**^1^** | R^2^ =0.98±0.026 | R^2^ =0.99±0.016 |

**NB**: **^1^**Each value is the average of three analyses ± standard deviation.
